# Supplementary material for: Icaritin protects SH-SY5Y cells transfected with TDP-43 by alleviating mitochondrial damage and oxidative stress
Source: PeerJ. 2021 Aug 10;9:e11978. doi: 10.7717/peerj.11978 (PMC8362678; doi:10.7717/peerj.11978)
Supplement: Supplemental Information 2 [file peerj-09-11978-s002.docx]

**Full-length uncropped blots**

**Materials and methods of Western blot assay**

Cells were collected, lysed with RIPA lysis buffer, and then centrifuged at 12,000 rpm at 4°C for 15 min. The total protein concentration was measured with a BCA protein assay kit (Beijing Solarbio). The proteins were then heated at 100°C for 5 min for denaturation. Equal amounts of total protein (25 μg per lane) were loaded on 12% SDS-PAGE gels and separated. After sample loading, the voltage was set to 90 V and then to 120 V until the sample reached the bottom of the gel. The proteins were then transferred to a nitrocellulose (NC) film by the sandwich method. The membrane was washed three times with TBST and then blocked with 5% skimmed milk. The membrane was then incubated for 12 h at 4°C with a GAPDH antibody (1:1000, Proteintech Group), TDP-43 antibody (1:1000, Proteintech Group), and CytC antibody (1:1000, Proteintech Group). The membrane was washed with TBST 3 times and incubated with HRP-conjugated Affinipure goat anti-rabbit lgG (H+L) (1:1000, Proteintech Group) for 2 h at room temperature. The membranes were developed using hydrogen peroxide and Supersignal West Pico Luminol (Pierce, Seymour Fisher Technologies). Finally, High-Sig ECL Western Blotting Substrate (Shanghai Tanon Technology Co., Ltd.) was used to visualize the membrane.

**Figure 1** Establishment of the TDP-43-transfected SH-SY5Y cell model.

**(E) Expression of TDP-43 in SH-SY5Y cells transfected with TDP-43.**


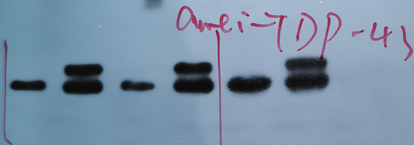


TDP-43-From left to right are Control, TDP-43, Control, TDP-43, Control, TDP-43 group.


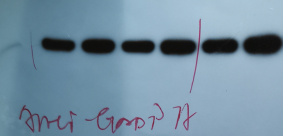


GAPDH-From left to right are Control, TDP-43, Control, TDP-43, Control, TDP-43 group.

**Figure 3** Influence of ICT treatment on TDP-43 expression in the control group compared with the TDP-43 group.


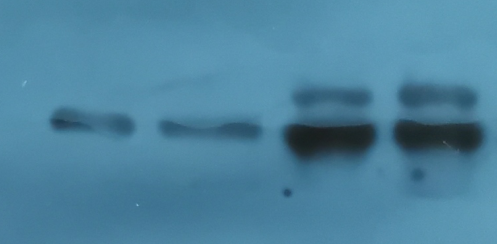


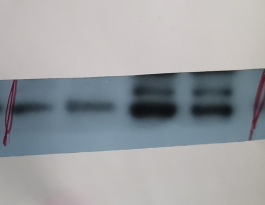


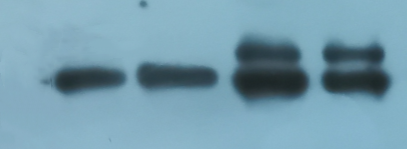


TDP-43-From left to right are Control, Control + ICT, TDP-43, TDP-43 + ICT group.


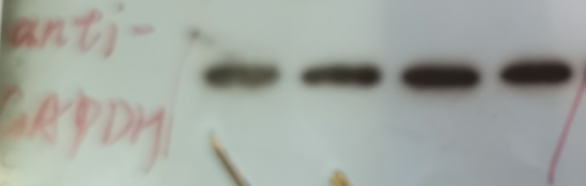


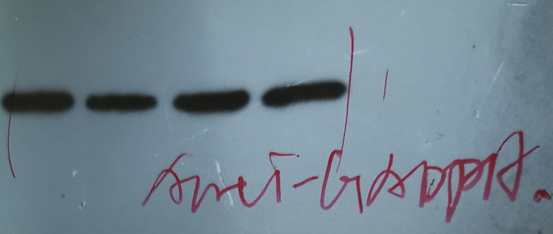

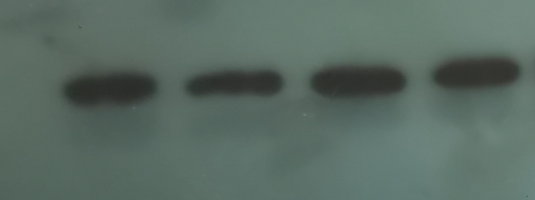


GAPDH-From left to right are Control, Control + ICT, TDP-43, TDP-43 + ICT group.

**Figure 4** Effects of ICT on the MMP, ATP content and CytC expression in TDP-43-transfected SH-SY5Y cells.

**(D) Influence of ICT on CytC expression in the model cells.**


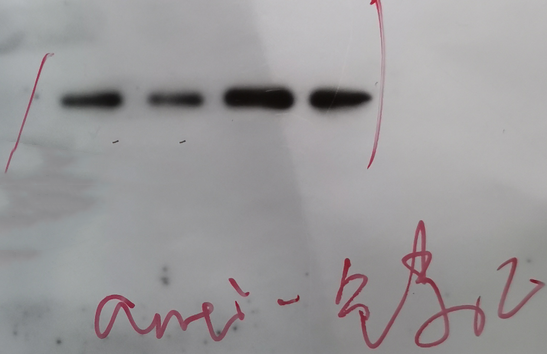


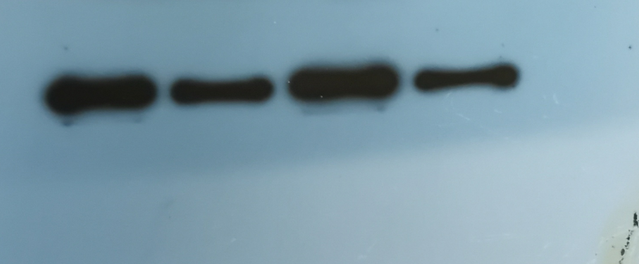


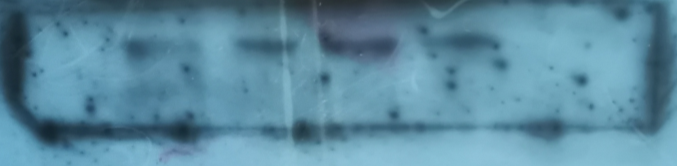


CytC-From left to right are Control, Control + ICT, TDP-43, TDP-43 + ICT group.


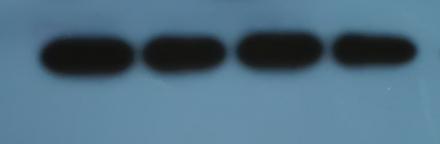


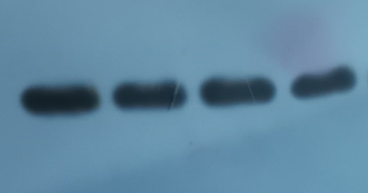


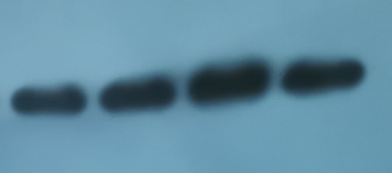


GAPDH-From left to right are Control, Control + ICT, TDP-43, TDP-43 + ICT group.
