# Supplementary material for: Icaritin protects SH-SY5Y cells transfected with TDP-43 by alleviating mitochondrial damage and oxidative stress
Source: PeerJ. 2021 Aug 10;9:e11978. doi: 10.7717/peerj.11978 (PMC8362678; doi:10.7717/peerj.11978)
Supplement: Supplemental Information 3 [file peerj-09-11978-s003.pdf]

TARDBP 过表达慢病毒载体构建流程及鉴定报告

一 实验材料

实验试剂

试剂名称	生产厂家
载体	Hanbio Biotechnology
大肠杆菌菌株 DH5α	Invitrogen
限制性内切酶	Thermo Fisher Scientific
HB-infusionTM 无缝克隆试剂盒	Hanbio Biotechnology Co., Ltd.
质粒 DNA 小，大量抽提试剂盒大	Beijing ComWin Biotech
凝胶回收试剂盒	Shanghai Generay Biotech
琼脂糖，琼脂粉	Sangon Biotech
DNA ladder	Thermo Fisher Scientific
KOD-Plus Kit	TOYOBO CO.,LTD.

载体及目的基因信息

1、pHBLV-CMV-MCS-3flag-EF1-puro 载体图谱如下：

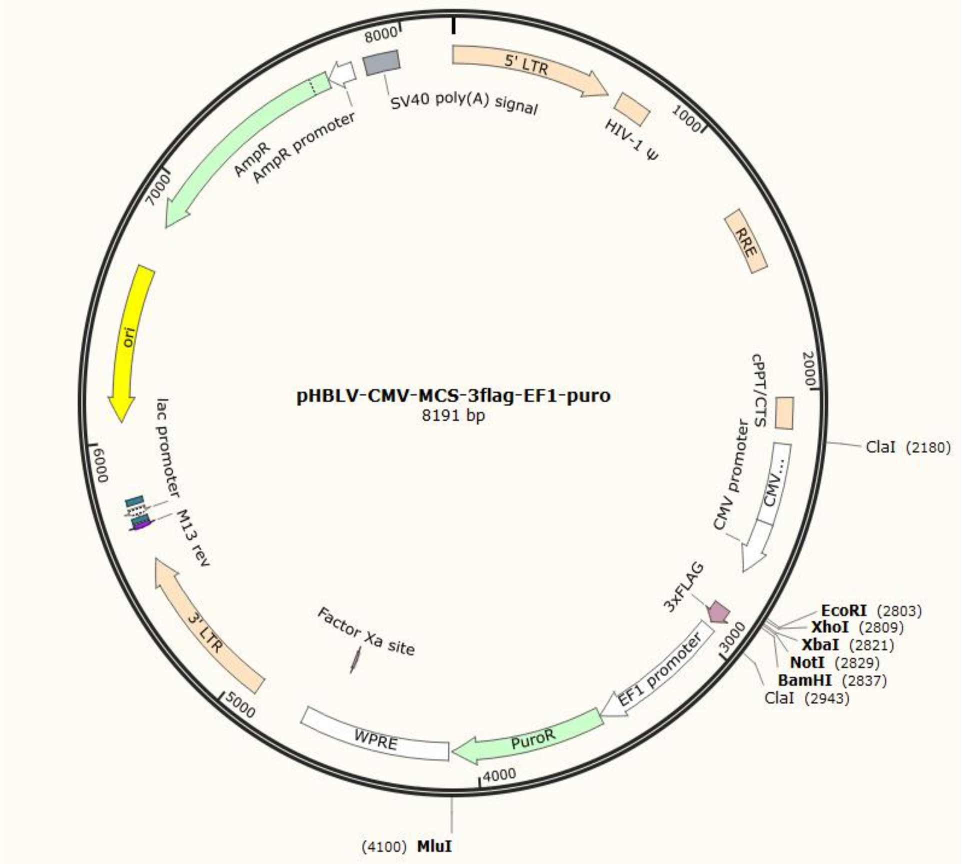

Tel：021-51296258
网址：http://www.hanbio.net
邮编号码：201203
公司地址：上海市浦东新区张江高科蔡伦路 150 号 1 号楼 2 楼 102
E-mail:service@hanbio.net

2、TARDBP 序列信息：

ATGTCTGAATATATTCGGGTAACCGAAGATGAGAACGATGAGCCCATTGAAATACCATCGGAAGACGATGGGACGGT
GCTGCTCTCCACGGTTACAGCCCAGTTTCCAGGGGCGTGTGGGCTTCGCTACAGGAATCCAGTGTCTCAGTGTATGA
GAGGTGTCCGGCTGGTAGAAGGAATTCTGCATGCCCCAGATGCTGGCTGGGGAAATCTGGTGTATGTTGTCAACTAT
CCAAAAGATAACAAAAGAAAAATGGATGAGACAGATGCTTCATCAGCAGTGAAAGTGAAAAGAGCAGTCCAGAAAAC
ATCCGATTTAATAGTGTTGGGTCTCCCATGGAAAACAACCGAACAGGACCTGAAAGAGTATTTTAGTACCTTTGGAG
AAGTTCTTATGGTGCAGGTCAAGAAAGATCTTAAGACTGGTCATTCAAAGGGGTTTGGCTTTGTTCGTTTTACGGAA
TATGAAACACAAGTGAAAGTAATGTCACAGCGACATATGATAGATGGACGATGGTGTGACTGCAAACTTCCTAATTC
TAAGCAAAGCCAAGATGAGCCTTTGAGAAGCAGAAAAGTGTTTGTGGGGCGCTGTACAGAGGACATGACTGAGGATG
AGCTGCGGGAGTTCTTCTCTCAGTACGGGGATGTGATGGATGTCTTCATCCCCAAGCCATTCAGGGCCTTTGCCTTT
GTTACATTTGCAGATGATCAGATTGCGCAGTCTCTTTGTGGAGAGGACTTGATCATTAAAGGAATCAGCGTTCATAT
ATCCAATGCCGAACCTAAGCACAATAGCAATAGACAGTTAGAAAGAAGTGGAAGATTTGGTGGTAATCCAGGTGGCT
TTGGGAATCAGGGTGGATTTGGTAATAGCAGAGGGGGTGGAGCTGGTTTGGGAAACAATCAAGGTAGTAATATGGGT
GGTGGGATGAACTTTGGTGCGTTCAGCATTAATCCAGCCATGATGGCTGCCGCCCAGGCAGCACTACAGAGCAGTTG
GGGTATGATGGGCATGTTAGCCAGCCAGCAGAACCAGTCAGGCCCATCGGGTAATAACCAAAACCAAGGCAACATGC
AGAGGGAGCCAAACCAGGCCTTCGGTTCTGGAAATAACTCTTATAGTGGCTCTAATTCTGGTGCAGCAATTGGTTGG
GGATCAGCATCCAATGCAGGGTCGGGCAGTGGTTTTAATGGAGGCTTTGGCTCAAGCATGGATTCTAAGTCTTCTGG
CTGGGGAATGTAG

引物设计：

TARDBP-F: atctatttccggtGAATTCCTCGAGATGTCTGAATATATTCGGGTAACCG

TARDBP-R: CTTAAGCTTGGTACCGAGGATCCCATTCCCCAGCCAGAAGACT

Tel：021-51296258
网址：http://www.hanbio.net
邮编号码：201203
公司地址：上海市浦东新区张江高科蔡伦路 150 号 1 号楼 2 楼 102
E-mail:service@hanbio.net

二、实验流程

1、载体 37℃酶切，胶回收
2、片段 PCR 之后回收
3、处理好的目的片段与载体连接，反应体系（20 μl）
4、转化 (感受态细胞: DH5α)，具体步骤见附录转化部分
5、抗性: Amp，37℃、230rpm/min，培养过夜
6、转化后的平板挑菌，37℃、230rpm/min 摇菌 14 小时，用菌液进行 PCR 鉴定，将阳性克隆菌液送测序公司测序
备注：详细实验流程及程序见附录 2

三、实验结果

1、TARDBP 过表达载体测序结果:

CTCGTTTAGTGACCGTCAGATCGCCTGGAGACGCCATCCACGCTGTTTTGACCTCCATAGAAGACACCGACTCTACT
AGAGGATCTATTTCCGGTGAATTCCTCGAGGCCACCATGTCTGAATATATTCGGGTAACCGAAGATGAGAACGATGA
GCCCATTGAAATACCATCGGAAGACGATGGGACGGTGCTGCTCTCCACGGTTACAGCCCAGTTTCCAGGGGCGTGTG
GGCTTCGCTACAGGAATCCAGTGTCTCAGTGTATGAGAGGTGTCCGGCTGGTAGAAGGAATTCTGCATGCCCCAGAT
GCTGGCTGGGGAAATCTGGTGTATGTTGTCAACTATCCAAAAGATAACAAAAGAAAAATGGATGAGACAGATGCTTC
ATCAGCAGTGAAAGTGAAAAGAGCAGTCCAGAAAACATCCGATTTAATAGTGTTGGGTCTCCCATGGAAAACAACCG
AACAGGACCTGAAAGAGTATTTTAGTACCTTTGGAGAAGTTCTTATGGTGCAGGTCAAGAAAGATCTTAAGACTGGT
CATTCAAAGGGGTTTGGCTTTGTTCGTTTTACGGAATATGAAACACAAGTGAAAGTAATGTCACAGCGACATATGAT
AGATGGACGATGGTGTGACTGCAAACTTCCTAATTCTAAGCAAAGCCAAGATGAGCCTTTGAGAAGCAGAAAAGTGT
TTGTGGGGCGCTGTACAGAGGACATGACTGAGGATGAGCTGCGGGAGTTCTTCTCTCAGTACGGGGATGTGATGGAT
GTCTTCATCCCCAAGCCATTCAGGGCCTTTGCCTTTGTTACATTTGCAGATGATCAGATTGCGCAGTCTCTTTGTGG
AGAGGACTTGATCATTAAAGGAATCAGCGTTCATATATCCAATGCCGAACCTAAGCACAATAGCAATAGACAGTTAG
AAAGAAGTGGAAGATTTGGTGGTAATCCAGGTGGCTTTGGGAATCAGGGTGGATTTGGTAATAGCAGAGGGGGTGGA
GCTGGTTTGGGAAACAATCAAGGTAGTAATATGGGTGGTGGGATGAACTTTGGTGCGTTCAGCATTAATCCAGCCAT
GATGGCTGCCGCCCAGGCAGCACTACAGAGCAGTTGGGGTATGATGGGCATGTTAGCCAGCCAGCAGAACCAGTCAG
GCCCATCGGGTAATAACCAAAACCAAGGCAACATGCAGAGGGAGCCAAACCAGGCCTTCGGTTCTGGAAATAACTCT
TATAGTGGCTCTAATTCTGGTGCAGCAATTGGTTGGGGATCAGCATCCAATGCAGGGTCGGGCAGTGGTTTTAATGG
AGGCTTTGGCTCAAGCATGGATTCTAAGTCTTCTGGCTGGGGAATGGGATCCTCGGTACCAAGCTTAAGTGACTACA
AGGATGACGATGACAAGGATTACAAAGACGACGATGATAAGGACTATAAGGATGATGACGACAAATAAAGATCCATC
GATACTAGTAAGGATCTGCGATCGCTCCGGTGCCCGTCAGTGGGCAGAGCGCACATCGCCCACAGTCCCCGAGAAGT
TGGGGGGAGGGGTCGGCAATTGAACGGGTGCCTAG

Tel：021-51296258
网址：http://www.hanbio.net
邮编号码：201203
公司地址：上海市浦东新区张江高科蔡伦路 150 号 1 号楼 2 楼 102
E-mail:service@hanbio.net

2、TARDBP 测序结果比对：（绿色区域为与目的序列匹配部分）

```
NoName           1 mseyirvtedendepieipseddgtvllstvtaqfpgacglryrnpvsqcmrgvrlvegilhapdagwgnlvyvvnypkdnkrkmdetdassavkvkrav
5.161v-h-TAR   114 mseyirvtedendepieipseddgtvllstvtaqfpgacglryrnpvsqcmrgvrlvegilhapdagwgnlvyvvnypkdnkrkmdetdassavkvkrav

NoName         301 qktsdlivlglpwktteqdlkeyfstfgevlmvqvkkdlktghskgfgfvrfteyetqvkvmsqrhmidgrwcdcklpnskqsqdeplrsrkvfvgrcte
5.161v-h-TAR   414 qktsdlivlglpwktteqdlkeyfstfgevlmvqvkkdlktghskgfgfvrfteyetqvkvmsqrhmidgrwcdcklpnskqsqdeplrsrkvfvgrcte

NoName         601 dmtedelreffsqygdvmdvfipkpfrafafvtfaddqiaqslcgedliikgisvhisnaepkhnsnrqlersgrfggnpggfgnqggfgnsrgggaglg
5.161v-h-TAR   714 dmtedelreffsqygdvmdvfipkpfrafafvtfaddqiaqslcgedliikgisvhisnaepkhnsnrqlersgrfggnpggfgnqggfgnsrgggaglg

NoName         901 nnqgsnmgggmnfgafsinpammaaaqaalqsswgmmgmlasqqnqsgpsgnnqnqgnmqrepnqafgsgnnsysgsnsgaaigwgsasnagsgsgfngg
5.161v-h-TAR  1014 nnqgsnmgggmnfgafsinpammaaaqaalqsswgmmgmlasqqnqsgpsgnnqnqgnmqrepnqafgsgnnsysgsnsgaaigwgsasnagsgsgfngg

NoName        1201 fgssmdskssgwgm-------------------------------------------------------------------------
5.161v-h-TAR  1314 fgssmdskssgwgmgssvpslsdykddddkdykddddkdykddddk[illegible]rsidtskdlrslrcpsvgrahiahsprevggrgrqlngcl
```

Tel：021-51296258
网址：http://www.hanbio.net
邮编号码：201203
公司地址：上海市浦东新区张江高科蔡伦路 150 号 1 号楼 2 楼 102
E-mail:service@hanbio.net

TARDBP overexpression lentiviral vector construction process and identification report

Materials:

Experimental reagent

Reagents	Manufacturer
vector	Hanbio Biotechnology
Escherichia coli strain DH5α	Invitrogen
Restriction endonuclease	Thermo Fisher Scientific
HB-infusionTM	Hanbio Biotechnology Co., Ltd.
Plasmid DNA extraction kit	Beijing ComWin Biotech
Gel Extraction Kit	Shanghai Generay Biotech
Agarose	Sangon Biotech
DNA ladder	Thermo Fisher Scientific
KOD-Plus Kit	TOYOBO CO.,LTD.

Vector and target gene information:

1. The vector map of pHBLV-CMV-MCS-3flag-EF1-puro is as follows:

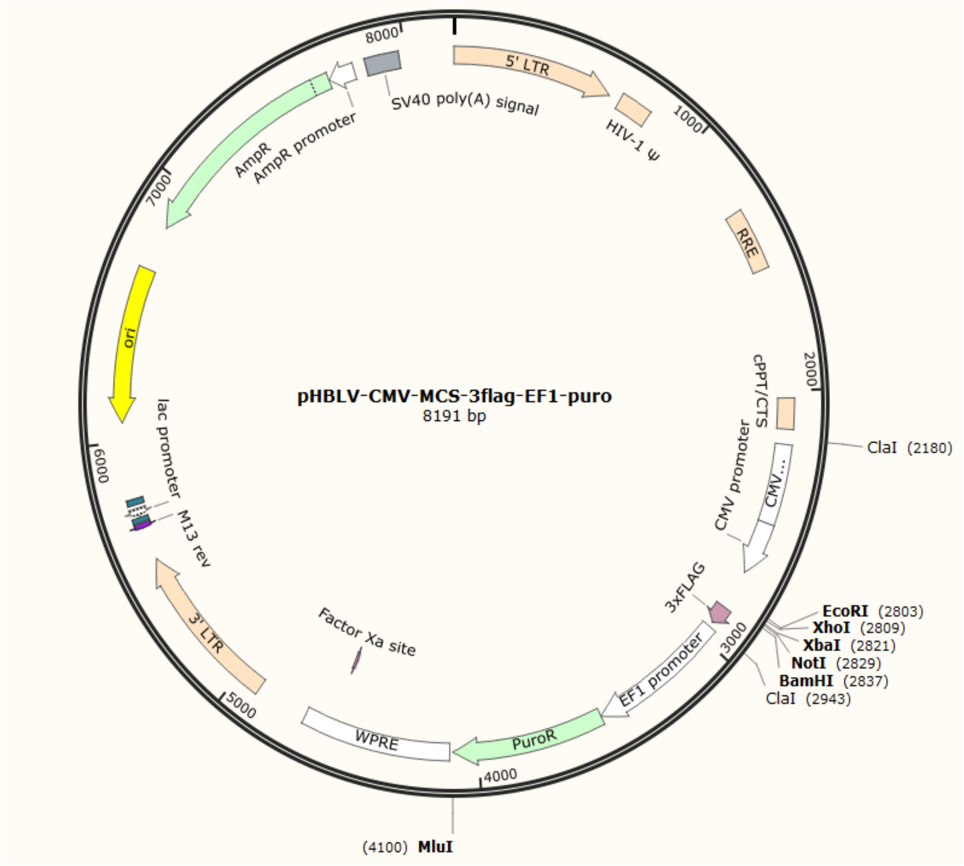

2. TARDBP sequence information:

ATGTCTGAATATATTCGGGTAACCGAAGATGAGAACGATGAGCCCATTGAAATACCATCGGAAGA
CGATGGGACGGTGCTGCTCTCCACGGTTACAGCCCAGTTTCCAGGGGCGTGTGGGCTTCGCTACA
GGAATCCAGTGTCTCAGTGTATGAGAGGTGTCCGGCTGGTAGAAGGAATTCTGCATGCCCCAGAT
GCTGGCTGGGGAAATCTGGTGTATGTTGTCAACTATCCAAAAGATAACAAAAGAAAAATGGATGA
GACAGATGCTTCATCAGCAGTGAAAGTGAAAAGAGCAGTCCAGAAAACATCCGATTTAATAGTGT
TGGGTCTCCCATGGAAAACAACCGAACAGGACCTGAAAGAGTATTTTAGTACCTTTGGAGAAGTT
CTTATGGTGCAGGTCAAGAAAGATCTTAAGACTGGTCATTCAAAGGGGTTTGGCTTTGTTCGTTT

TACGGAATATGAAACACAAGTGAAAGTAATGTCACAGCGACATATGATAGATGGACGATGGTGTG
ACTGCAAACTTCCTAATTCTAAGCAAAGCCAAGATGAGCCTTTGAGAAGCAGAAAAGTGTTTGTG
GGGCGCTGTACAGAGGACATGACTGAGGATGAGCTGCGGGAGTTCTTCTCTCAGTACGGGGATGT
GATGGATGTCTTCATCCCCAAGCCATTCAGGGCCTTTGCCTTTGTTACATTTGCAGATGATCAGA
TTGCGCAGTCTCTTTGTGGAGAGGACTTGATCATTAAAGGAATCAGCGTTCATATATCCAATGCC
GAACCTAAGCACAATAGCAATAGACAGTTAGAAAGAAGTGGAAGATTTGGTGGTAATCCAGGTGG
CTTTGGGAATCAGGGTGGATTTGGTAATAGCAGAGGGGGTGGAGCTGGTTTGGGAAACAATCAAG
GTAGTAATATGGGTGGTGGGATGAACTTTGGTGCGTTCAGCATTAATCCAGCCATGATGGCTGCC
GCCCAGGCAGCACTACAGAGCAGTTGGGGTATGATGGGCATGTTAGCCAGCCAGCAGAACCAGTC
AGGCCCATCGGGTAATAACCAAAACCAAGGCAACATGCAGAGGGAGCCAAACCAGGCCTTCGGTT
CTGGAAATAACTCTTATAGTGGCTCTAATTCTGGTGCAGCAATTGGTTGGGGATCAGCATCCAAT
GCAGGGTCGGGCAGTGGTTTTAATGGAGGCTTTGGCTCAAGCATGGATTCTAAGTCTTCTGGCTG
GGGAATGTAG

Primer design:

TARDBP-F： atctatttccggtGAATTCCTCGAGATGTCTGAATATATTCGGGTAACCG

TARDBP-R： CTTAAGCTTGGTACCGAGGATCCCATTCCCCAGCCAGAAGACT

Methods：

1. Carriers are digested at 37°C, and glue is recovered
2. Recover fragments after PCR
3. The processed target fragment is connected to the carrier, and the reaction system (20 μl)
4. Transformation (competent cells: DH5α), see the transformation section in the appendix for specific steps
5. Resistance: Amp, 37°C, 230rpm/min, culture overnight
6. Pick the bacteria on the transformed plate, shake the bacteria for 14 hours at 37°C, 230rpm/min, use the bacteria solution for PCR identification, and send the positive clone bacteria solution to the sequencing company for sequencing

Remarks: Please refer to appendix 2 for detailed experimental procedures and procedures

Results：

1.TARDBP overexpression vector sequencing results:

CTCGTTTAGTGACCGTCAGATCGCCTGGAGACGCCATCCACGCTGTTTTGACCTCCATA
GAAGACACCGACTCTACTAGAGGATCTATTTCCGGTGAATTCCTCGAGGCCACCATGTC
TGAATATATTCGGGTAACCGAAGATGAGAACGATGAGCCCATTGAAATACCATCGGAAG
ACGATGGGACGGTGCTGCTCTCCACGGTTACAGCCCAGTTTCCAGGGGCGTGTGGGCT
TCGCTACAGGAATCCAGTGTCTCAGTGTATGAGAGGTGTCCGGCTGGTAGAAGGAATT
CTGCATGCCCCAGATGCTGGCTGGGGAAATCTGGTGTATGTTGTCAACTATCCAAAAGA
TAACAAAAGAAAAATGGATGAGACAGATGCTTCATCAGCAGTGAAAGTGAAAAGAGC
AGTCCAGAAAACATCCGATTTAATAGTGTTGGGTCTCCCATGGAAAACAACCGAACAG
GACCTGAAAGAGTATTTTAGTACCTTTGGAGAAGTTCTTATGGTGCAGGTCAAGAAAG
ATCTTAAGACTGGTCATTCAAAGGGGTTTGGCTTTGTTCGTTTTACGGAATATGAAACA
CAAGTGAAAGTAATGTCACAGCGACATATGATAGATGGACGATGGTGTGACTGCAAAC
TTCCTAATTCTAAGCAAAGCCAAGATGAGCCTTTGAGAAGCAGAAAAGTGTTTGTGGG
GCGCTGTACAGAGGACATGACTGAGGATGAGCTGCGGGAGTTCTTCTCTCAGTACGGG

GATGTGATGGATGTCTTCATCCCCAAGCCATTCAGGGCCTTTGCCTTTGTTACATTTGCA
GATGATCAGATTGCGCAGTCTCTTTGTGGAGAGGACTTGATCATTAAAGGAATCAGCGT
TCATATATCCAATGCCGAACCTAAGCACAATAGCAATAGACAGTTAGAAAGAAGTGGA
AGATTTGGTGGTAATCCAGGTGGCTTTGGGAATCAGGGTGGATTTGGTAATAGCAGAG
GGGGTGGAGCTGGTTTGGGAAACAATCAAGGTAGTAATATGGGTGGTGGGATGAACTT
TGGTGCGTTCAGCATTAATCCAGCCATGATGGCTGCCGCCCAGGCAGCACTACAGAGC
AGTTGGGGTATGATGGGCATGTTAGCCAGCCAGCAGAACCAGTCAGGCCCATCGGGTA
ATAACCAAAACCAAGGCAACATGCAGAGGGAGCCAAACCAGGCCTTCGGTTCTGGAA
ATAACTCTTATAGTGGCTCTAATTCTGGTGCAGCAATTGGTTGGGGATCAGCATCCAATG
CAGGGTCGGGCAGTGGTTTTAATGGAGGCTTTGGCTCAAGCATGGATTCTAAGTCTTCT
GGCTGGGGAATGGGATCCTCGGTACCAAGCTTAGTGACTACAAGGATGACGATGACA
AGGATTACAAAGACGACGATGATAAGGACTATAAGGATGATGACGACAAATAAAGATC
CATCGATACTAGTAAGGATCTGCGATCGCTCCGGTGCCCGTCAGTGGGCAGAGCGCAC
ATCGCCCACAGTCCCCGAGAAGTTGGGGGGAGGGGTCGGCAATTGAACGGTGCCTA
G

2.Comparison of TARDBP sequencing results: (The green area is the part that matches the target sequence):

```
NoName           1 mseyirvtedendepieipseddgtvllstvtaqfpgacglryrnpvsqcmrgvrlvegilhapdagwgnlvyvvnypkdnkrkmdetdassavkvkrav
5.16lv-h-TAR   114 mseyirvtedendepieipseddgtvllstvtaqfpgacglryrnpvsqcmrgvrlvegilhapdagwgnlvyvvnypkdnkrkmdetdassavkvkrav

NoName         301 qktsdlivlglpwktteqdlkeyfstfgevlmvqvkkdlktghskgfgfvrfteyetqvkvmsqrhmidgrwcdcklpnskqsqdeplrsrkvfvgrcte
5.16lv-h-TAR   414 qktsdlivlglpwktteqdlkeyfstfgevlmvqvkkdlktghskgfgfvrfteyetqvkvmsqrhmidgrwcdcklpnskqsqdeplrsrkvfvgrcte

NoName         601 dmtedelreffsqygdvmdvfipkpfrafafvtfaddqiaqslcgedliikgisvhisnaepkhnsnrqlersgrfggnpggfgnqggfgnsrgggaglg
5.16lv-h-TAR   714 dmtedelreffsqygdvmdvfipkpfrafafvtfaddqiaqslcgedliikgisvhisnaepkhnsnrqlersgrfggnpggfgnqggfgnsrgggaglg

NoName         901 nnqgsnmgggmnfgafsinpammaaaqaalqsswgmmgmlasqqnqsgpsgnnqnqgnmqrepnqafgsgnnsysgsnsgaaigwgsasnagsgsgfngg
5.16lv-h-TAR  1014 nnqgsnmgggmnfgafsinpammaaaqaalqsswgmmgmlasqqnqsgpsgnnqnqgnmqrepnqafgsgnnsysgsnsgaaigwgsasnagsgsgfngg

NoName        1201 fgssmdskssgwgm-------------------------------------------------------------------------
5.16lv-h-TAR  1314 fgssmdskssgwgmgssvpslsdykddddkdykddddkdykddddk*rsidtskdlrslrcpsvgrahiahsprevggrgrqlngcl
```

慢病毒包装实验报告

一、慢病毒包装流程

制备慢病毒穿梭质粒及其辅助包装原件载体质粒，三种质粒载体分别进行高纯度无内毒素抽提，共转染 293T 细胞，转染后 6 h 更换为完全培养基，培养 48 h 和 72 h 后，收集富含慢病毒颗粒的细胞上清液，4°C，2000 × g ，10 min，去除细胞碎片，然后收集病毒上清液，利用超离：4°C，82700 × g，离心 120 min，对其超离，最后得到高滴度的慢病毒超离液。

1.实验材料

1.1 实验试剂

试剂名称	生产厂家	产品货号
胎牛血清	Thermo	26050070
大肠杆菌菌株 DH5α	Tiangen	CB101-03
胰蛋白酶	Thermo	LP0042
质粒 DNA 小,大量抽提试剂盒	Tiangen	DP117
DMEM	Thermo	11965118
Lipofiter^{TM}	Hanbio Biotechnology	HB-TRCF-1000
PBS	Thermo	10010001

真核转染试剂为汉恒生物(汉恒生物，Hanbio）产品

1.2 慢病毒载体、包装细胞和菌株

病毒包装系统：

三质粒系统，pSPAX2、pMD2G 和穿梭质粒（携带目的基因或者 shRNA）。

包装细胞株：293T，慢病毒的包装细胞，为贴壁依赖型成上皮样细胞，生长培养基为 DMEM（含 10 % FBS）。贴壁细胞经培养生长增殖形成单层细胞。

菌株：大肠杆菌菌株 DH5-α，用于扩增慢病毒载体和辅助包装载体质粒。

Tel：021-51296258
网址：http://www.hanbio.net
邮编号码：201203
公司地址：上海市浦东新区张江高科蔡伦路 150 号 1 号楼 2 楼 102
E-mail:service@hanbio.net

2、实验目的

使用三质粒包装系统获得高滴度的慢病毒颗粒。

3、实验步骤

3.1 质粒扩增

构建好的慢病毒载体和辅助质粒需要大量抽提，浓度大于 1 μg/μL，A260/280 在 1.7~1.8 之间方可用以包毒。

3.2 病毒包装

第一天：铺板 293T 细胞用于转染（前提是细胞已经培养到传代后可以满足后续转染实验需要）。操作完毕后置于 37° C，5 % CO_2 和 95 % 相对湿度的培养箱中。

第三天：转染

1、观察细胞密度，达到 70~80 % 的汇合率即可进行转染。

2、做脂转 complex：Opti MEM 需在 37° C 水浴中预热，Lipofiter^{TM} 转染试剂需恢复至室温方可使用，使用前需摇匀。

转染每皿 100mm 的 complex 成分如下：

pSPAX2	10 μg
pMD2G	5 μg
穿梭质粒	10 μg
Lipofiter^{TM} 用量	75 μL

3、转染后更换含 10 % 胎牛血清 FBS 的新鲜完全培养基，如果在当天上午进行转染，转染后 6 h 进行换液，如果当天下午进行转染，转染后第二天早上约转染后 16 h 进行换液。

4、收毒：转染后 48 h 和 72 h 分别两次收集病毒上清（48 h 收集后置换新鲜完全培养基）。在 48 h 收毒时，将 100 mm dish 中的培养基倒入 50 mL 离心管中，注意培养皿壁不要接触离心管口，以防出现细菌污染，随后补入 10 mL 含 10 % 胎牛血清 FBS 的新鲜完全培养基，平稳置于 37° C，5 % CO_2 的恒温培养箱中继续培养。在 72 h 收毒时，直接将 100 mm dish 中的培养基倒入 50 mL 离心管中，同样注意培养皿壁不要接触离心管口，以防出现细菌污染。收完 72 h 的病毒原液后，该 dish 便可以舍弃。

Tel：021-51296258
网址：http://www.hanbio.net
邮编号码：201203
公司地址：上海市浦东新区张江高科蔡伦路 150 号 1 号楼 2 楼 102
E-mail:service@hanbio.net

5、超速离心：将 50 mL 离心管中的病毒上清，4° C ，2000 × g ，离心 10 min，去除细胞碎片；然后收集病毒原液上清置于超速离心管中，4° C ，82700 × g ，离心 120 min，最后将慢病毒超离液分装到灭菌处理的病毒管中。

6、病毒保存：按照要求分装病毒，标记（病毒名称，年-月-日），-80° C 冰箱保存。

Tel：021-51296258
网址：http://www.hanbio.net
邮编号码：201203
公司地址：上海市浦东新区张江高科蔡伦路 150 号 1 号楼 2 楼 102
E-mail:service@hanbio.net

Lentivirus packaging experiment report

Lentivirus Packaging and Production

Prepare the lentiviral shuttle plasmid and its auxiliary packaging original vector plasmids. The three plasmid vectors were extracted with high purity and endotoxin-free, and were co-transfected into 293T cells. After 6 hours of transfection, they were replaced with complete medium and cultured for 48 hours and 72 hours. Then, collect the cell supernatant rich in lentiviral particles, 4°C, 2000 × g, 10 min, remove the cell debris, then collect the virus supernatant, use ultra-isolation: 4°C, 82700 × g, centrifuge 120 min, the ultra-isolation of it, and finally a high-titer lentivirus ultra-isolation.

1.Materials：

Reagents	Manufacturer	product number
Fetal Bovine Serum	Thermo	26050070
Escherichia coli strain DH5α	Tiangen	CB101-03
Trypsin	Thermo	LP0042
Plasmid DNA extraction kit	Tiangen	DP117
DMEM	Thermo	11965118
Lipofiter^{TM}	Hanbio Biotechnology	HB-TRCF-1000
PBS	Thermo	10010001

1.2 Lentiviral vectors, packaging cells and strains

Virus packaging system:

Three plasmid system, pSPAX2, pMD2G and shuttle plasmid (carrying target gene or shRNA).

Packaging cell line: 293T, lentivirus packaging cells, which are anchorage-dependent epithelial-like cells, and the growth medium is DMEM (containing 10% FBS). Adherent cells grow and proliferate through culture to form a monolayer of cells.

Strain: Escherichia coli strain DH5-α, used to amplify lentiviral vector and auxiliary packaging vector plasmid.

2. Purpose

A three-plasmid packaging system is used to obtain high-titer lentiviral particles.

3. Experimental steps

3.1 Plasmid amplification

The constructed lentiviral vector and helper plasmid need a large amount of extraction, the concentration is greater than 1 μg/μL, and the A260/280 is between 1.7 and 1.8 before it can be used to encapsulate the virus.

3.2 Virus packaging

Day 1: Plating 293T cells for transfection (provided that the cells have been cultured to passaging to meet the needs of subsequent transfection experiments). After the operation, place it in an incubator at 37°C, 5% CO_2 and 95% relative humidity.

Day 3: Transfection

1. Observe the cell density and perform transfection after reaching a confluency of 70~80%.

2. Do liposuction complex: Opti MEM needs to be preheated in a 37°C water bath, and Lipofiter^{TM} transfection reagent needs to be restored to room temperature before use. Shake well before use.

4. After transfection, change the fresh complete medium containing 10% fetal bovine serum FBS. If transfection is carried out in the morning, change the medium 6 hours after transfection. If transfection is carried out in the afternoon, the next morning after transfection The medium was changed about 16 h after transfection.
5. Collection of the virus: the virus supernatant was collected twice at 48 h and 72 h after transfection (replaced with fresh complete medium after 48 h collection). When the poison is collected for 48 hours, pour the medium in the 100 mm dish into a 50 mL centrifuge tube, and pay attention to the wall of the petri dish not to touch the opening of the centrifuge tube to prevent bacterial contamination, and then add 10 mL containing 10% fetal bovine serum FBS fresh complete medium, stably placed in a constant temperature incubator at 37°C and 5% CO_2 to continue culturing. When the poison is collected within 72 hours, directly pour the medium in the 100 mm dish into a 50 mL centrifuge tube, and also pay attention to the wall of the petri dish not to touch the centrifuge tube opening to prevent bacterial contamination. After receiving the 72-hour virus stock, the dish can be discarded.
6. Ultracentrifugation: Centrifuge the virus supernatant in a 50 mL centrifuge tube at 2000 × g at 4°C for 10 min to remove cell debris; then collect the virus stock supernatant and place it in an ultracentrifuge tube at 4°C, 82700 × g, centrifuge for 120 min, and finally aliquot the lentivirus into sterile virus tubes.
7. Virus preservation: install the virus according to the requirements, mark (virus name, year-month-day), and store in the refrigerator at -80°C.
